# 2-Chloro­ethyl 4-nitro­benzoate

**DOI:** 10.1107/S1600536810038742

**Published:** 2010-10-02

**Authors:** Hao Wu, Min-Hao Xie, Pei Zou, Ya-Ling Liu, Yong-Jun He

**Affiliations:** a Key Laboratory of Nuclear Medicine, Ministry of Health, Jiangsu Key Laboratory of Molecular Nuclear Medicine, Jiangsu Institute of Nuclear Medicine, Wuxi 214063, People’s Republic of China

## Abstract

The title compound, C_9_H_8_ClNO_4_, crystallizes with two mol­ecules in the asymmetric unit. In each mol­ecule, the carboxyl­ate group is nearly coplanar with the benzene ring, forming dihedral angles of 2.4 (1) and 4.9 (1)°. In the crystal, mol­ecules are linked through weak C—H⋯O and C—H⋯Cl hydrogen bonds. A short O⋯N contact of 2.7660 (19) Å occurs between the nitro groups of adjacent mol­ecules.

## Related literature

For benzoates as inter­mediates in the chemistry of pigments and pharmaceuticals, see: Zhang *et al.* (1995 ▶, 1990 ▶). For a related structure, see: Wu *et al.* (2009 ▶).
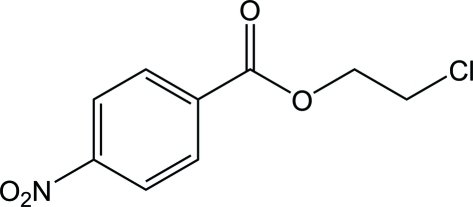


## Experimental

### 

#### Crystal data

C_9_H_8_ClNO_4_
*M* *_r_* = 229.61
Monoclinic, 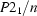

*a* = 4.9404 (10) Å
*b* = 21.618 (5) Å
*c* = 18.325 (4) Å
β = 90.441 (3)°
*V* = 1957.0 (7) Å^3^
*Z* = 8
Mo *K*α radiation
μ = 0.38 mm^−1^
*T* = 103 K
0.40 × 0.20 × 0.20 mm

#### Data collection

Rigaku SPIDER diffractometer
Absorption correction: multi-scan (*ABSCOR*; Higashi, 1995 ▶) *T*_min_ = 0.862, *T*_max_ = 0.927
18618 measured reflections
4466 independent reflections
3781 reflections with *I* > 2σ(*I*)
*R*_int_ = 0.033

#### Refinement

*R*[*F* ^2^ > 2σ(*F* ^2^)] = 0.034
*wR*(*F* ^2^) = 0.084
*S* = 1.00
4466 reflections
271 parameters
H-atom parameters constrained
Δρ_max_ = 0.34 e Å^−3^
Δρ_min_ = −0.23 e Å^−3^

### 

Data collection: *RAPID-AUTO* (Rigaku, 2004 ▶); cell refinement: *RAPID-AUTO*; data reduction: *RAPID-AUTO*; program(s) used to solve structure: *SHELXTL* (Sheldrick, 2008 ▶); program(s) used to refine structure: *SHELXTL*; molecular graphics: *SHELXTL*; software used to prepare material for publication: *SHELXTL*.

## Supplementary Material

Crystal structure: contains datablocks I, global. DOI: 10.1107/S1600536810038742/xu5038sup1.cif

Structure factors: contains datablocks I. DOI: 10.1107/S1600536810038742/xu5038Isup2.hkl

Additional supplementary materials:  crystallographic information; 3D view; checkCIF report

## Figures and Tables

**Table 1:** Hydrogen-bond geometry (Å, °)

*D*—H⋯*A*	*D*—H	H⋯*A*	*D*⋯*A*	*D*—H⋯*A*
C1—H1⋯Cl1′	0.95	2.79	3.6234 (18)	147
C2′—H2′⋯O3′^i^	0.95	2.50	3.318 (2)	145
C4′—H4′⋯O2	0.95	2.40	3.223 (2)	144
C5—H5⋯O2′^ii^	0.95	2.42	3.205 (2)	140
C9—H9*B*⋯O2′^iii^	0.99	2.55	3.526 (2)	167

Symmetry codes: (i) 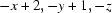; (ii) 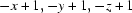; (iii) 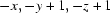.

**Fig. 1.**
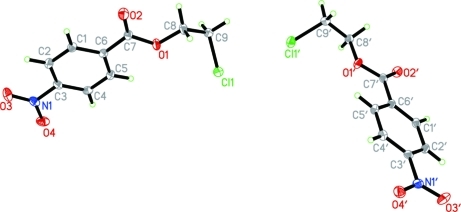
eA view of the title compound with the atomic numbering scheme. Displacement ellipsoids were drawn at the 50% probability level.

## supplementary crystallographic information

### Comment

Benzoates are important intermediates in the chemistry of pigments and pharmaceuticals, which are widely used all over the world (Zhang *et al.*, 1995; Zhang *et al.*, 1990). The crystall structure of Methyl 4-nitrobenzoate has been reported (Wu *et al.*, 2009). As an extension of our study, we report here the crystal structure of the title compound.

The asymmetric unit of the title compound (Fig.1) contains two crystallographically independent molecules in an asymmetric unit.The crystal data show that the bond lengths and angles are within expected ranges. The ester groups and the benzene rings in the two molecules are almost coplanar, as indicated by the dihedral angles of 2.4 (1)° (O1/O2/C6/C7; C1—C6) and 4.9 (1)° (O1'/O2'/C6'/C7'; C1'—C6'). The dihedral angle between the ring(C1—C6) and the ring(C1'—C6') is 92.7 (1)°.

In the cyrstal structure, adjacent molecules are linked together by the weak C—H···O and C—H···Cl hydrogen bonds (Table 1). Intramolecular C—H···O interactions are also observed. These hydrogen-bonding interactions stabilize the crystal structure.

### Experimental

Commercial 2-chloroethyl 4-nitrobenzoate was recrystallized by slow evaporation of methanol/water solution (1:1 v/v). Colourless single crystals were formed after two weeks.

### Refinement

H atoms were placed in calculated positions with C–H = 0.99 (methylene) and 0.95 Å (aromatic), and were refined in a riding mode with U_iso_(H) = 1.2U_eq_(C).

### Crystal data

**Table:**

C_9_H_8_ClNO_4_	*F*(000) = 944
*M**_r_* = 229.61	*D*_x_ = 1.559 Mg m^−^^3^
Monoclinic, *P*2_1_/*n*	Mo *K*α radiation, λ = 0.71073 Å
Hall symbol: -P 2yn	Cell parameters from 5688 reflections
*a* = 4.9404 (10) Å	θ = 3.0–27.5°
*b* = 21.618 (5) Å	µ = 0.38 mm^−^^1^
*c* = 18.325 (4) Å	*T* = 103 K
β = 90.441 (3)°	Prism, colorless
*V* = 1957.0 (7) Å^3^	0.40 × 0.20 × 0.20 mm
*Z* = 8	

### Data collection

**Table:**

Rigaku SPIDER diffractometer	4466 independent reflections
Radiation source: Rotating Anode	3781 reflections with *I* > 2σ(*I*)
graphite	*R*_int_ = 0.033
ω scans	θ_max_ = 27.5°, θ_min_ = 3.0°
Absorption correction: multi-scan (*ABSCOR*; Higashi, 1995)	*h* = −6→6
*T*_min_ = 0.862, *T*_max_ = 0.927	*k* = −28→28
18618 measured reflections	*l* = −23→20

### Refinement

**Table:**

Refinement on *F*^2^	Primary atom site location: structure-invariant direct methods
Least-squares matrix: full	Secondary atom site location: difference Fourier map
*R*[*F*^2^ > 2σ(*F*^2^)] = 0.034	Hydrogen site location: inferred from neighbouring sites
*wR*(*F*^2^) = 0.084	H-atom parameters constrained
*S* = 1.00	*w* = 1/[σ^2^(*F*_o_^2^) + (0.0441*P*)^2^ + 0.680*P*] where *P* = (*F*_o_^2^ + 2*F*_c_^2^)/3
4466 reflections	(Δ/σ)_max_ = 0.001
271 parameters	Δρ_max_ = 0.34 e Å^−^^3^
0 restraints	Δρ_min_ = −0.23 e Å^−^^3^

### Special details

**Table:**

Geometry. All e.s.d.'s (except the e.s.d. in the dihedral angle between two l.s. planes) are estimated using the full covariance matrix. The cell e.s.d.'s are taken into account individually in the estimation of e.s.d.'s in distances, angles and torsion angles; correlations between e.s.d.'s in cell parameters are only used when they are defined by crystal symmetry. An approximate (isotropic) treatment of cell e.s.d.'s is used for estimating e.s.d.'s involving l.s. planes.
Refinement. Refinement of *F*^2^ against ALL reflections. The weighted *R*-factor *wR* and goodness of fit *S* are based on *F*^2^, conventional *R*-factors *R* are based on *F*, with *F* set to zero for negative *F*^2^. The threshold expression of *F*^2^ > σ(*F*^2^) is used only for calculating *R*-factors(gt) *etc*. and is not relevant to the choice of reflections for refinement. *R*-factors based on *F*^2^ are statistically about twice as large as those based on *F*, and *R*- factors based on ALL data will be even larger.

### Fractional atomic coordinates and isotropic or equivalent isotropic displacement parameters (Å^2^)

**Table:**

	*x*	*y*	*z*	*U*_iso_*/*U*_eq_	
Cl1	0.58062 (8)	0.615621 (18)	0.60156 (2)	0.02137 (10)	
O1	0.5719 (2)	0.70861 (5)	0.72719 (6)	0.0201 (2)	
O2	0.4652 (2)	0.80061 (5)	0.77677 (6)	0.0230 (3)	
O3	1.4451 (3)	0.74075 (6)	1.03965 (7)	0.0288 (3)	
O4	1.5555 (2)	0.65746 (6)	0.98123 (7)	0.0274 (3)	
N1	1.4158 (3)	0.70367 (6)	0.98970 (8)	0.0203 (3)	
C1	0.8669 (3)	0.78260 (7)	0.88589 (9)	0.0169 (3)	
H1	0.7699	0.8205	0.8865	0.020*	
C2	1.0633 (3)	0.77121 (7)	0.93827 (9)	0.0176 (3)	
H2	1.1025	0.8007	0.9752	0.021*	
C3	1.2012 (3)	0.71558 (7)	0.93535 (8)	0.0163 (3)	
C4	1.1480 (3)	0.67097 (7)	0.88291 (9)	0.0169 (3)	
H4	1.2455	0.6331	0.8825	0.020*	
C5	0.9499 (3)	0.68282 (7)	0.83117 (8)	0.0163 (3)	
H5	0.9090	0.6528	0.7949	0.020*	
C6	0.8102 (3)	0.73879 (7)	0.83215 (8)	0.0143 (3)	
C7	0.5974 (3)	0.75381 (7)	0.77687 (8)	0.0155 (3)	
C8	0.3759 (3)	0.71808 (7)	0.66929 (9)	0.0193 (3)	
H8A	0.4555	0.7434	0.6299	0.023*	
H8B	0.2148	0.7399	0.6882	0.023*	
C9	0.2977 (3)	0.65561 (7)	0.64066 (9)	0.0183 (3)	
H9A	0.1541	0.6605	0.6031	0.022*	
H9B	0.2231	0.6305	0.6810	0.022*	
Cl1'	0.09925 (8)	0.578510 (18)	0.46926 (2)	0.02083 (10)	
O1'	0.0719 (2)	0.53366 (5)	0.30930 (6)	0.0170 (2)	
O2'	−0.0224 (2)	0.45272 (5)	0.23673 (6)	0.0203 (2)	
O3'	0.9692 (2)	0.56670 (5)	0.00593 (7)	0.0249 (3)	
O4'	1.0593 (2)	0.63815 (6)	0.08520 (6)	0.0243 (3)	
N1'	0.9309 (3)	0.59294 (6)	0.06415 (7)	0.0176 (3)	
C1'	0.3955 (3)	0.49087 (7)	0.13991 (8)	0.0158 (3)	
H1'	0.3083	0.4527	0.1291	0.019*	
C2'	0.5964 (3)	0.51288 (7)	0.09445 (9)	0.0165 (3)	
H2'	0.6497	0.4903	0.0525	0.020*	
C3'	0.7174 (3)	0.56882 (7)	0.11200 (8)	0.0150 (3)	
C4'	0.6481 (3)	0.60310 (7)	0.17254 (9)	0.0170 (3)	
H4'	0.7366	0.6411	0.1831	0.020*	
C5'	0.4468 (3)	0.58079 (7)	0.21743 (8)	0.0160 (3)	
H5'	0.3941	0.6037	0.2592	0.019*	
C6'	0.3209 (3)	0.52453 (7)	0.20138 (8)	0.0139 (3)	
C7'	0.1063 (3)	0.49883 (7)	0.24947 (8)	0.0149 (3)	
C8'	−0.1343 (3)	0.51265 (7)	0.35872 (8)	0.0168 (3)	
H8'1	−0.0718	0.4753	0.3852	0.020*	
H8'2	−0.3017	0.5022	0.3314	0.020*	
C9'	−0.1878 (3)	0.56394 (8)	0.41134 (9)	0.0211 (3)	
H9'1	−0.3454	0.5530	0.4418	0.025*	
H9'2	−0.2334	0.6020	0.3839	0.025*	

### Atomic displacement parameters (Å^2^)

**Table:**

	*U*^11^	*U*^22^	*U*^33^	*U*^12^	*U*^13^	*U*^23^
Cl1	0.01863 (19)	0.0242 (2)	0.0213 (2)	−0.00012 (15)	0.00069 (15)	−0.00381 (16)
O1	0.0238 (6)	0.0183 (5)	0.0182 (6)	0.0036 (5)	−0.0087 (5)	−0.0042 (4)
O2	0.0287 (6)	0.0174 (5)	0.0228 (6)	0.0070 (5)	−0.0078 (5)	−0.0007 (5)
O3	0.0304 (7)	0.0329 (7)	0.0229 (7)	−0.0062 (5)	−0.0103 (6)	0.0002 (5)
O4	0.0203 (6)	0.0359 (7)	0.0259 (7)	0.0089 (5)	0.0000 (5)	0.0089 (5)
N1	0.0156 (6)	0.0272 (7)	0.0182 (7)	−0.0039 (6)	−0.0011 (5)	0.0071 (6)
C1	0.0191 (8)	0.0141 (7)	0.0174 (8)	0.0020 (6)	0.0006 (6)	−0.0007 (6)
C2	0.0209 (8)	0.0178 (7)	0.0142 (8)	−0.0020 (6)	0.0001 (6)	−0.0011 (6)
C3	0.0131 (7)	0.0215 (7)	0.0144 (7)	−0.0020 (6)	−0.0004 (6)	0.0050 (6)
C4	0.0165 (7)	0.0150 (7)	0.0191 (8)	0.0026 (6)	0.0021 (6)	0.0020 (6)
C5	0.0199 (8)	0.0142 (7)	0.0148 (7)	−0.0005 (6)	0.0013 (6)	−0.0012 (6)
C6	0.0157 (7)	0.0146 (7)	0.0127 (7)	−0.0006 (6)	0.0015 (6)	0.0016 (6)
C7	0.0179 (7)	0.0155 (7)	0.0131 (7)	−0.0016 (6)	0.0009 (6)	0.0005 (6)
C8	0.0217 (8)	0.0192 (7)	0.0168 (8)	0.0010 (6)	−0.0071 (6)	−0.0008 (6)
C9	0.0152 (7)	0.0209 (7)	0.0189 (8)	−0.0011 (6)	0.0006 (6)	−0.0018 (6)
Cl1'	0.0243 (2)	0.02306 (19)	0.01513 (19)	−0.00621 (15)	0.00131 (15)	−0.00093 (15)
O1'	0.0212 (6)	0.0176 (5)	0.0123 (5)	−0.0048 (4)	0.0038 (4)	−0.0015 (4)
O2'	0.0235 (6)	0.0157 (5)	0.0216 (6)	−0.0044 (5)	0.0024 (5)	−0.0019 (5)
O3'	0.0313 (7)	0.0233 (6)	0.0203 (6)	0.0061 (5)	0.0107 (5)	0.0013 (5)
O4'	0.0200 (6)	0.0302 (6)	0.0225 (6)	−0.0072 (5)	−0.0001 (5)	0.0030 (5)
N1'	0.0154 (6)	0.0206 (7)	0.0167 (7)	0.0054 (5)	0.0004 (5)	0.0052 (5)
C1'	0.0183 (7)	0.0139 (7)	0.0153 (8)	0.0010 (6)	−0.0024 (6)	−0.0015 (6)
C2'	0.0184 (7)	0.0178 (7)	0.0134 (7)	0.0047 (6)	−0.0009 (6)	−0.0025 (6)
C3'	0.0125 (7)	0.0181 (7)	0.0144 (7)	0.0026 (6)	−0.0001 (6)	0.0048 (6)
C4'	0.0183 (8)	0.0164 (7)	0.0164 (8)	−0.0014 (6)	−0.0022 (6)	0.0002 (6)
C5'	0.0195 (8)	0.0154 (7)	0.0130 (7)	0.0000 (6)	0.0002 (6)	−0.0021 (6)
C6'	0.0146 (7)	0.0145 (7)	0.0127 (7)	0.0014 (6)	−0.0021 (6)	0.0013 (6)
C7'	0.0164 (7)	0.0144 (7)	0.0138 (7)	0.0031 (6)	−0.0026 (6)	0.0006 (6)
C8'	0.0170 (7)	0.0200 (7)	0.0134 (8)	−0.0038 (6)	0.0032 (6)	0.0012 (6)
C9'	0.0172 (8)	0.0261 (8)	0.0202 (8)	0.0018 (6)	0.0014 (6)	−0.0025 (7)

### Geometric parameters (Å, °)

**Table:**

Cl1—C9	1.7971 (16)	Cl1'—C9'	1.7927 (17)
O1—C7	1.3408 (18)	O1'—C7'	1.3419 (18)
O1—C8	1.4454 (19)	O1'—C8'	1.4416 (18)
O2—C7	1.2043 (18)	O2'—C7'	1.2041 (18)
O3—N1	1.2245 (19)	O3'—N1'	1.2243 (18)
O4—N1	1.2247 (18)	O4'—N1'	1.2258 (17)
N1—C3	1.472 (2)	N1'—C3'	1.4725 (19)
C1—C2	1.382 (2)	C1'—C2'	1.385 (2)
C1—C6	1.393 (2)	C1'—C6'	1.393 (2)
C1—H1	0.9500	C1'—H1'	0.9500
C2—C3	1.383 (2)	C2'—C3'	1.386 (2)
C2—H2	0.9500	C2'—H2'	0.9500
C3—C4	1.385 (2)	C3'—C4'	1.380 (2)
C4—C5	1.381 (2)	C4'—C5'	1.382 (2)
C4—H4	0.9500	C4'—H4'	0.9500
C5—C6	1.393 (2)	C5'—C6'	1.396 (2)
C5—H5	0.9500	C5'—H5'	0.9500
C6—C7	1.490 (2)	C6'—C7'	1.491 (2)
C8—C9	1.498 (2)	C8'—C9'	1.494 (2)
C8—H8A	0.9900	C8'—H8'1	0.9900
C8—H8B	0.9900	C8'—H8'2	0.9900
C9—H9A	0.9900	C9'—H9'1	0.9900
C9—H9B	0.9900	C9'—H9'2	0.9900
			
C7—O1—C8	117.01 (12)	C7'—O1'—C8'	115.53 (11)
O3—N1—O4	124.40 (14)	O3'—N1'—O4'	124.11 (14)
O3—N1—C3	118.16 (14)	O3'—N1'—C3'	118.11 (13)
O4—N1—C3	117.44 (14)	O4'—N1'—C3'	117.79 (13)
C2—C1—C6	120.41 (14)	C2'—C1'—C6'	120.14 (14)
C2—C1—H1	119.8	C2'—C1'—H1'	119.9
C6—C1—H1	119.8	C6'—C1'—H1'	119.9
C1—C2—C3	118.13 (14)	C1'—C2'—C3'	118.03 (14)
C1—C2—H2	120.9	C1'—C2'—H2'	121.0
C3—C2—H2	120.9	C3'—C2'—H2'	121.0
C2—C3—C4	122.76 (14)	C4'—C3'—C2'	123.12 (14)
C2—C3—N1	118.58 (14)	C4'—C3'—N1'	118.16 (13)
C4—C3—N1	118.65 (14)	C2'—C3'—N1'	118.72 (14)
C5—C4—C3	118.48 (14)	C3'—C4'—C5'	118.36 (14)
C5—C4—H4	120.8	C3'—C4'—H4'	120.8
C3—C4—H4	120.8	C5'—C4'—H4'	120.8
C4—C5—C6	120.04 (14)	C4'—C5'—C6'	120.00 (14)
C4—C5—H5	120.0	C4'—C5'—H5'	120.0
C6—C5—H5	120.0	C6'—C5'—H5'	120.0
C1—C6—C5	120.18 (14)	C1'—C6'—C5'	120.34 (14)
C1—C6—C7	118.00 (13)	C1'—C6'—C7'	118.48 (13)
C5—C6—C7	121.83 (14)	C5'—C6'—C7'	121.17 (14)
O2—C7—O1	124.27 (14)	O2'—C7'—O1'	123.57 (14)
O2—C7—C6	124.32 (14)	O2'—C7'—C6'	124.79 (14)
O1—C7—C6	111.41 (12)	O1'—C7'—C6'	111.64 (12)
O1—C8—C9	107.38 (12)	O1'—C8'—C9'	107.52 (12)
O1—C8—H8A	110.2	O1'—C8'—H8'1	110.2
C9—C8—H8A	110.2	C9'—C8'—H8'1	110.2
O1—C8—H8B	110.2	O1'—C8'—H8'2	110.2
C9—C8—H8B	110.2	C9'—C8'—H8'2	110.2
H8A—C8—H8B	108.5	H8'1—C8'—H8'2	108.5
C8—C9—Cl1	111.97 (11)	C8'—C9'—Cl1'	111.67 (11)
C8—C9—H9A	109.2	C8'—C9'—H9'1	109.3
Cl1—C9—H9A	109.2	Cl1'—C9'—H9'1	109.3
C8—C9—H9B	109.2	C8'—C9'—H9'2	109.3
Cl1—C9—H9B	109.2	Cl1'—C9'—H9'2	109.3
H9A—C9—H9B	107.9	H9'1—C9'—H9'2	107.9
			
C6—C1—C2—C3	0.3 (2)	C6'—C1'—C2'—C3'	−0.3 (2)
C1—C2—C3—C4	−0.8 (2)	C1'—C2'—C3'—C4'	0.4 (2)
C1—C2—C3—N1	178.39 (13)	C1'—C2'—C3'—N1'	−179.86 (13)
O3—N1—C3—C2	7.2 (2)	O3'—N1'—C3'—C4'	−171.12 (13)
O4—N1—C3—C2	−171.95 (14)	O4'—N1'—C3'—C4'	8.8 (2)
O3—N1—C3—C4	−173.58 (14)	O3'—N1'—C3'—C2'	9.1 (2)
O4—N1—C3—C4	7.3 (2)	O4'—N1'—C3'—C2'	−170.90 (13)
C2—C3—C4—C5	0.4 (2)	C2'—C3'—C4'—C5'	−0.6 (2)
N1—C3—C4—C5	−178.79 (13)	N1'—C3'—C4'—C5'	179.68 (13)
C3—C4—C5—C6	0.5 (2)	C3'—C4'—C5'—C6'	0.6 (2)
C2—C1—C6—C5	0.5 (2)	C2'—C1'—C6'—C5'	0.4 (2)
C2—C1—C6—C7	−179.66 (14)	C2'—C1'—C6'—C7'	−179.20 (13)
C4—C5—C6—C1	−0.9 (2)	C4'—C5'—C6'—C1'	−0.5 (2)
C4—C5—C6—C7	179.26 (14)	C4'—C5'—C6'—C7'	179.00 (14)
C8—O1—C7—O2	1.3 (2)	C8'—O1'—C7'—O2'	−0.4 (2)
C8—O1—C7—C6	−178.60 (13)	C8'—O1'—C7'—C6'	179.34 (12)
C1—C6—C7—O2	−2.3 (2)	C1'—C6'—C7'—O2'	−5.3 (2)
C5—C6—C7—O2	177.53 (15)	C5'—C6'—C7'—O2'	175.17 (15)
C1—C6—C7—O1	177.65 (13)	C1'—C6'—C7'—O1'	174.96 (13)
C5—C6—C7—O1	−2.5 (2)	C5'—C6'—C7'—O1'	−4.60 (19)
C7—O1—C8—C9	−156.81 (13)	C7'—O1'—C8'—C9'	−166.85 (12)
O1—C8—C9—Cl1	−62.71 (15)	O1'—C8'—C9'—Cl1'	−66.44 (15)

### Hydrogen-bond geometry (Å, °)

**Table:**

*D*—H···*A*	*D*—H	H···*A*	*D*···*A*	*D*—H···*A*
C1—H1···Cl1'^i^	0.95	2.79	3.6234 (18)	147
C2'—H2'···O3'^ii^	0.95	2.50	3.318 (2)	145
C4'—H4'···O2^iii^	0.95	2.40	3.223 (2)	144
C5—H5···O2'^iv^	0.95	2.42	3.205 (2)	140
C5'—H5'···O1'	0.95	2.39	2.7112 (19)	100
C9—H9B···O2'^v^	0.99	2.55	3.526 (2)	167

Symmetry codes: (i) ; (ii) −*x*+2, −*y*+1, −*z*; (iii) ; (iv) −*x*+1, −*y*+1, −*z*+1; (v) −*x*, −*y*+1, −*z*+1.
